# Iron Redox Speciation Analysis Using Capillary Electrophoresis Coupled to Inductively Coupled Plasma Mass Spectrometry (CE-ICP-MS)

**DOI:** 10.3389/fchem.2019.00136

**Published:** 2019-03-14

**Authors:** Bernhard Michalke, Desiree Willkommen, Vivek Venkataramani

**Affiliations:** ^1^Research Unit Analytical BioGeoChemistry, Helmholz Zentrum München, German Research Center for Environmental Health, Neuherberg, Germany; ^2^Department of Hematology and Medical Oncology, University Medical Center Göttingen (UMG), Göttingen, Germany; ^3^Institute of Pathology, University Medical Center Göttingen (UMG), Göttingen, Germany

**Keywords:** iron redox speciation analysis, capillary electrophoresis, inductively-coupled-plasma-mass-spectrometry, neurodegeneration, brain research, ferroptosis

## Abstract

Neuronal iron dyshomeostasis occurs in multiple neurodegenerative diseases. Changes in the Fe(II)/Fe(III) ratio toward Fe(II) is closely related to oxidative stress, lipid peroxidation, and represents a hallmark feature of ferroptosis. In particular for body fluids, like cerebrospinal fluid (CSF), reliable quantitative methods for Fe(II)/(III) redox-speciation analysis are needed to better assess the risk of Fe(II)-mediated damage in brain tissue. Currently in the field of metallomics, the most direct method to analyze both iron species is via LC-ICP-MS. However, this Fe(II)/(III) speciation analysis method suffers from several limitations. Here, we describe a unique method using capillary electrophoresis (CE)-ICP-MS for quantitative Fe(II)/(III) speciation analysis that can be applied for cell lysates and biofluid samples. Compared to LC, CE offers various advantages: (1) Capillaries have no stationary phase and do not depend on batch identity of stationary phases; (2) Replacement of aged or blocked capillaries is quick with no performance change; (3) Purge steps are effective and short; (4) Short sample analysis time. The final method employed 20 mM HCl as background electrolyte and a separation voltage of +25 kV. In contrary to the LC-method, no complexation of Fe-species with pyridine dicarboxylic acid (PDCA) was applied, since it hampered separation. Peak shapes and concentration detection limits were improved by combined conductivity-pH-stacking achieving 3 μg/L detection limit (3σ) at 13 nL injection volume. Calibrations from LOD—150 μg/L were linear [*r*^2^_[Fe(II)]_ = 0.9999, *r*^2^_[Fe(III)]_ = 0.9951]. At higher concentrations Fe(II) curve flattened significantly. Measurement precision was 3.5% [Fe(II) at 62 μg/L] or 2.2% [Fe(III) at 112 μg/L] and migration time precision was 2% for Fe(III) and 3% for Fe(II), each determined in 1:2 diluted lysates of human neuroblastoma cells. Concentration determination accuracy was checked by parallel measurements of SH-SY5Y cell lysates with validated LC-ICP-MS method and by recovery experiments after standard addition. Accuracy (*n* = 6) was 97.6 ± 3.7% Fe(III) and 105 ± 6.6%Fe(II). Recovery [*(a)* +33 μg/L or *(b)* +500 μg/L, addition per species] was (*a*): 97.2 ± 13% [Fe(II)], 108 ± 15% [Fe(III)], 102.5 ± 7% (sum of species), and *(b)* 99±4% [Fe(II)], 101 ± 6% [Fe(III)], 100 ± 5% (sum of species). Migration time shifts in CSF samples were due to high salinity, but both Fe-species were identified by standard addition.

## Introduction

Nowadays within neurodegeneration research it is most evident that iron-mediated oxidative stress (OS) and lipid peroxidation (LPO) plays a crucial role in multiple neurodegenerative brain disorders, such as Alzheimer's and Parkinson's disease (Hare et al., 2015, 2018; Ashraf et al., 2018). In brain, OS and LPO are closely related to the state of the redox-couple Fe(II)/Fe(III). While Fe(III) is redox-inactive, Fe(II) potently generates reactive oxygen species (ROS) via catalyzing the decomposition of H_2_O_2_, that results in highly toxic hydroxyl radicals and membrane LPO via Haber-Weiss and Fenton reactions (Kehrer, 2000; Sies, 2015; Gaschler and Stockwell, 2017). Excess of Fe(II)-generated ROS and peroxidized phospholipids are hampering the integrity of proteins, lipids, and DNA on a cellular level (Michalke et al., 2009; Solovyev, 2015), decreasing neuronal functions (Sies, 2015), and even are capable to trigger iron-dependent programmed necrotic cell death, known as “ferroptosis” (FPT) (Dixon et al., 2012; Stockwell et al., 2017). Therefore, methods that quantitatively analyze Fe(II)/(III) redox speciation in cell and tissue lysates and in biofluids are of eminent importance in brain and neurodegeneration research.

Chemical speciation analysis is a well-established tool to study the biological role and metabolism of trace elements in general (Michalke et al., 2009; Vinceti et al., 2013) as well in neurodegeneration (Michalke et al., 2007b; Fernsebner et al., 2014; Neth, 2015; Neth et al., 2015; Venkataramani et al., 2018; Willkommen et al., 2018). In our previous study, we revealed that liquid chromatography-coupled-to-inductively-coupled plasma mass spectrometry (LC-ICP-MS) represents a suitable Fe(II)/Fe(III) speciation analysis method that provides good figures of merit using only 8–10 min analysis time per sample (Solovyev et al., 2017). However, in routine LC work we observed several issues. Depending of the type of samples, excessive purge times were needed between runs. Moreover, when replacement of LC-columns becomes necessary after analyzing a big sample size, new LC columns for iron speciation analysis turned out to vary from batch-to-batch with changed performance and consequently needed time-consuming re-optimizations of elution conditions. These problems hampered high-throughput analysis and took additional time to gain acceptable result reliability. This prompted us to seek for analytical alternatives being less dependent on stationary phase batch uniformity of chromatographic columns.

Capillary electrophoresis (CE) is a well-suited analytical technique that uses an electrical field to separate ions based on their electrophoretic mobility (Thiebault and Dovichi, 1998). We developed a CE-ICP-MS based method for the quantification of Fe(II)/Fe(III) redox species because of several advantages of CE over other techniques such as LC: (1) Capillaries have no stationary phase and thus depend (nearly) not on batch identity; (2) Aged or blocked CE columns can be replaced quickly without altering performance; (3) Purge steps between samples are effective and quick resulting in a shorter analysis time per sample. We finally discuss the figures of merit of this Fe(II)/Fe(III) speciation analysis method that are currently accepted to be useful for application to pre-clinical samples such as human dopaminergic neuroblastoma cell lysates as well as clinical samples e.g., cerebrospinal fluid (CSF) samples (Iliff et al., 2012).

To our best knowledge, we here report for the first time the stepwise development of a CE-ICP-MS based method for Fe(II)/(III) speciation analysis, application and method performance to representative pre- and clinical samples. Due to the short analysis time and simplicity to “regenerate” the capillary we propose the superiority to alternative LC-ICP-MS-based methods.

## Experimental

### Chemicals

Tetramethylammoniumhydroxide (TMAH), HCl suprapure, and ammonium citrate were purchased from Merck (Darmstadt, Germany). Argon_liqud_ (Ar) was purchased from Air-Liquide (Düsseldorf, Germany) and gaseous Ar was gained at the vaporizer at the tank. FeCl_3_ · 6H_2_O standard, 2,6-pyridine dicarboxylic acid (dipicolinic acid, PDCA) and Manganese-acetate [Mn (II)] was from Sigma Aldrich Chemie (Steinheim, Germany). FeCl_2_ · 4H_2_O was purchased from AppliChem GmbH (Darmstadt, Germany). Sodium deoxycholate, NP-40, MnCl_2_ · 4H_2_O, phenylmethane sulfonyl fluoride or phenylmethylsulfonyl fluoride (PMSF), fetal calf serum, glutamine, phosphate buffered saline (PBS) and orthovanadate were purchased from Sigma-Aldrich (Taufkirchen, Germany). Dulbecco's modified Eagle's medium (DMEM) was delivered from Thermo Fisher Scientific (München, Germany). The cOmplete™ Protease Inhibitor Cocktail was from Roche, Mannheim, Germany. Chemicals were of highest available purity, i.e., all chemicals in the speciation laboratory were bought in ultrapure quality. All solutions were prepared by using MilliQ® water (18.2 mΩcm, Merck-Millipore, Darmstadt, Germany).

### Samples and Sample Preparation for Capillary Electrophoresis

Ar overlay in sample containers and immediate deep freezing after aliquoting had been applied for our samples and standard.

### Preparation of Standards

Stock solutions of FeCl_2_· 4H_2_O and FeCl_3_ · 6H_2_O were prepared by exact weighing respective amounts of standards (powder) into Falcon® tubes and dissolving in 10 mL MilliQ® water (concentration of stock solutions: 100 mg Fe/L). Stock solutions were aliquoted, overlaid with Ar and stored at −20°C. Working standards (e.g., 30 μg/L, or 100 μg/L) were prepared daily from an aliquot of freshly thawed stock standard by appropriate dilution with MilliQ® water and stored at +4°C.

### Preparation of SH-SY5Y Cell Lysates

In this paper, cell lysates from the human neuroblastoma cell line SH-SY5Y served as Fe(II)/(III)-relevant bio-matrix to show the performance and reliability of the new developed method. They were taken from parallel running experiments detailed in Venkataramani et al. (2018). In brief, SH-SY5Y was initially purchased from ATCC and validated for the heterozygous ALK p.F1174L mutation by Sanger sequencing. Cells were cultured in DMEM (Dulbecco's modified Eagle's medium) supplemented with 10% (v/v) fetal calf serum, 1% L-glutamine and 1% penicillin/streptomycin. Complete media were changed every 2–3 days. After reaching 70–80% confluency, cells were incubated with 100 μM Mn(II) dissolved in OptiMEM or left untreated (OptiMEM alone) for 24 h, washed twice with PBS and scraped into modified radioimmunoprecipitation assay (RIPA) lysis buffer (PBS) pH 7.4, 0.5% sodium deoxycholate, 1% NP-40 containing 1 mM PMSF, 1 mM orthovanadate, and 1x cOmplete™ Protease Inhibitor on ice for 45 min with gentle agitation. Cell lysates were centrifuged at 10,000 × g for 10 min and supernatants were sent on dry ice to the speciation laboratory in Helmholtz Center Munich.

### CSF Samples

CSF samples were taken from parallel running experiments detailed in Willkommen et al. (2018). In brief, CSF samples were taken by standardized lumbar puncture at Cologne University Hospital. After lumbar puncture and clinical chemistry analysis samples were stored at −80°C and finally sent on dry ice to the speciation laboratory in Helmholtz Center Munich. This study was approved by the Ethics Committee of the University Cologne (09.12.2014, no. 14-364) and all patients consented to the scientific use of their CSF samples.

### Instruments and Instrumental Conditions

#### Capillary Zone Electrophoresis (CZE)

A “PrinCe 706” CE system from PrinCe Technologies B.V. (Emmen, The Netherlands) was employed. Temperature settings for sample/buffer tray and capillary were set at 20°C by air cooling, each. The capillary (CS-Chromatographie Service GmbH, Langerwehe, Germany) for hyphenation to the ICP-MS was uncoated with dimension 85 cm × 50 μm ID. Before each run, the capillary was purged with 1 M TMAH and subsequently with 0.2% HCl and background electrolyte (each step 1 min, 4 bar, see also Table 1). Our first speciation analysis approach was derived from LC eluents (Solovyev et al., 2017) serving as electrolytes, where 50 mM ammonium citrate, 7.0 mM PDCA, pH = 4.2 was the optimal condition to keep redox species stabilized and provided good separation for the ionic-Fe(II)/(III)-PDCA complexes by cation exchange chromatography. This electrolyte served as background electrolyte in capillary, in inlet vial and as outlet/sheath electrolyte at CE-ICP-MS interface and separation voltage was set to + 25 kV. Since clear separation of the Fe(II)-PDCA complex from Fe(III)-PDCA complex was not achieved in CZE using those conditions, but further preliminary experiments showed promising separation with simple acidic eluents, subsequent experiments used electrolytes with diluted HCl. Finally, the following separation method was employed, using pH- and conductivity stacking (with leading and terminating electrolyte) for improved focusing of Fe-redox-species (see Table 1):

**Table 1:** Final method for Fe(II)/Fe(III) speciation analysis.

**Step-No**	**Step**	**Chemical**	**Condition**
**A**	**Preparation of CE column**		
A1	Capillary cleaning	1 M TMAH	4 bar, 1 min.
A2	Capillary conditioning	0.2% HCl	4 bar, 1 min.
A3	Capillary purging with background electrolyte	20 mM HCl	4 bar, 1 min.
A4	Stacking: leading electrolyte	1 M TMAH	150 mbar, 3 s
A5	Injection	Sample	150 mbar, 3 s
A6	Stacking: terminating electrolyte	0.05 mM HCl	150 mbar, 3 s
**B**	**Analysis**		
	Inlet electrolyte	20 mM HCl	150 mbar, 5 min, + 25 kV
	Outlet/sheath electrolyte at ICP-MS interface	5 mM HCl	Interface self-aspiration ca. 100 μL/min

*Section A: capillary preparation steps before measurement; Section B: electrolytes and conditions during analysis*.

#### Coupling of CZE to the ICP-MS

A CE-ICP-MS interface was installed based on a micro-mist nebulizer (100 μL), which was fitting into a homemade spray chamber. For coupling of the CE to the ICP-MS, a T-piece made from polyethylene was installed. The right end of the timber of the “T” was mounted at the nebulizer while the CE capillary entered at its left end, moving through the timber and ending at the nebulizer capillary. In analogy to our previous work, the positioning of the CE capillary was not critical with respect to signal response and stability (Michalke, 2004). Due to the nebulizer's aspiration, an auxiliary flow was introduced via the bottom-arm of the T-piece, flowing coaxially around the CE capillary to the nebulizer. Another T-piece was installed in the auxiliary flow line for introducing the grounded outlet electrode into the sheath electrolyte flow. This set-up provided the electrical connection between CE capillary end and outlet electrode. The self-aspiration mode allowed for best flow rate adjustment and avoided suction flow. Diluted hydrochloric acid (5 mM) was used as the auxiliary liquid.

#### Inductively Coupled Plasma Mass Spectrometry (ICP-MS) as CE Detector

A NexIon 300 D (Perkin Elmer, Sciex, Toronto, Canada) was operated as ICP-MS system for the on-line detection of CE-efflux. The isotopes ^56^Fe and ^57^Fe were measured in dynamic reaction cell (DRC) mode. The RF power was set to 1,250 W, the plasma gas was 16 L Ar/min. The nebulizer gas was optimized and finally set to 0.98 L Ar/min. The dwell time was 50 ms. Ammonia was used as DRC gas (0.58 ml NH_3_/min) and DRC rejection parameter was set to 0.58.

#### Software

The capillary electrophoresis system worked with the CE-system DAx-3D operation software from PrinCe, the NexIon ICP-MS operated with Syngistix operation software from Perkin Elmer. Syngistix software provided Fe-electropherogram files which were directly processed with PeakFit™ software Version 4.12, a non-linear curve fitting software.

## Results and Discussion

**Figure 1:**
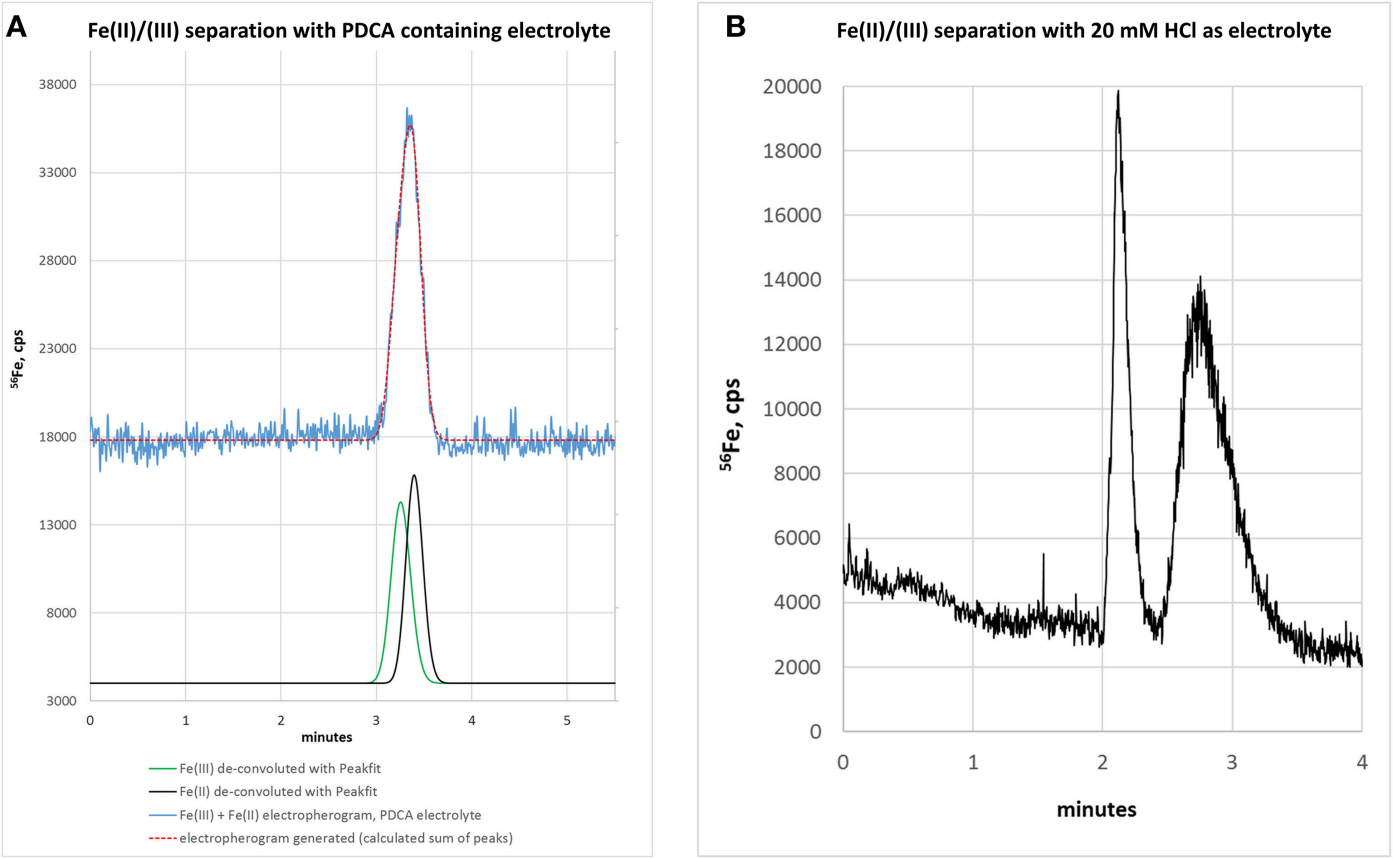
**(A)** Electropherogram of a standard mixture [Fe(II) + Fe(III)], each 100 μg/L, with ammonium citrate—PDCA electrolyte. Separation is not achieved. Mathematical peak deconvolution shows that the peak consist of two compounds (green, black) with similar migration times and the calculated sum-peak of both (red) matches the measured signal (blue). For graphical reasons the blue and red lines are plotted with an offset. **(B)** Electropherogram of Fe(III) and Fe(II) with 20 mM HCl as background electrolyte. Separation is possible but peak focusing is still insufficient. Therefore, sample stacking was planned for the next method development step.

Based on our previous experience with LC-ICP-MS, we first started with a 50 mM ammonium citrate electrolyte containing 7 mM PDCA, pH 4.2 (Solovyev et al., 2017). We here used the chelating agent PDCA, since it was previously reported to improve separation and species stability in LC-based speciation analysis approaches (Chen et al., 2012). We revealed that the PDCA addition to electrolyte markedly improved the peak shape when analyzing single standards of Fe(II) or Fe(III). However, migration times differed only slightly, indicating that no clear separation of both species could be achieved. The confirmation of this supposition is shown in Figure 1A where a mixture of both standards was analyzed (each 100 μg/L). While the electropherogram presented a broadened peak with maximum at 3.38 min (blue trace), the Fe(II) standard could not be discriminated from the Fe(III) standard. In this regard, we applied a peak deconvolution algorithm to separate out the contribution of each overlapping peak. PeakFit^TM^ software calculated two compounds for this peak with 49.9% of peak area for Fe(III)—corresponding to 93 μg/L [deconvoluted peak area related to Fe(III) calibration]—and 50.1% for Fe(II)—corresponding to 112 μg/L [deconvoluted peak area related to Fe(II) calibration]. Moreover, subsequent experiments with ammonium citrate and addition 0, 3, or 10 mM PDCA provided no satisfactory resolution of peaks.

**Figure 2:**
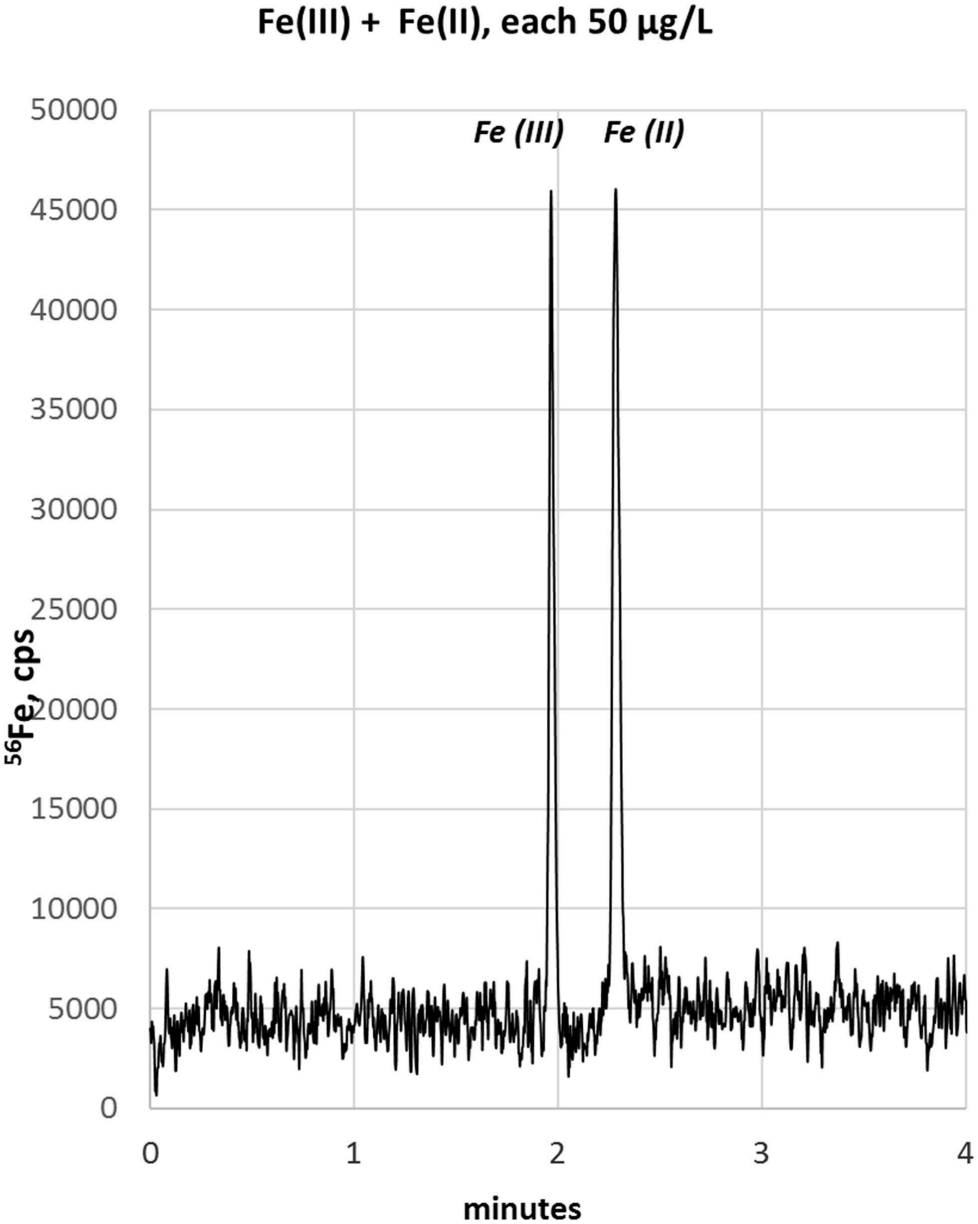
Electropherogram of 50 μg/L standards using the optimized conditions.

In order to improve the focusing in the positive voltage mode of CZE, we followed an approach with a lower pH. Indeed, a simple electrolyte with 20 mM HCl provided very good separation, short runs below 4 min and comparatively low current varying between 3 and 10 μA. The latter has some importance, since increased current up to 150 μA, as observed with e.g., 70 mM HCl concentration, resulted in capillary heating. Especially when analyzing bio-samples, elevated temperatures not only cause protein clogging inside the capillary, but also result in the reduction of heme-associated iron from the ferric to the ferrous state (Richards, 2009). The starting experiments with 20 mM HCl as electrolyte were promising and showed acceptable separation of the two iron species, however, peak focusing was not optimal. As presented in Figure 1B, Fe(III) peak (100 μg/L) appeared at about 2 min migration time, showed a peak width at baseline (PW_b_) of 24 s and reached a net-peak height (nPH) of only 17,000 cps. Fe(II) (200 μg/L) performed even worse with a PW_b_ of 60 s achieving a nPH of just 11,000 cps. According to the limited peak focusing, the compounds are just baseline separated with wide instead of high peaks. This would worsen detection limits and separation capability in real-life bio-samples. When analyte's concentration is too small (i.e., <LOD) and/or peak height vs. peak width needs to be improved, it is possible to place electrolytes with very low vs. very high conductivity or low vs. high pH before vs. behind the increased sample plug volume inside the capillary. This method, known as stacking, increases the total amount of analyte ions (using increased sample volume) and parallel effects focusing of the ions at pH- or conductivity-borders: Because the sample plug has a lower concentration of buffer ions, the effective field strength across the sample plug is larger than that in the rest of the capillary. As a result, cations in the sample plug migrate toward the cathode with a greater velocity and the anions migrate more slowly. When the ions reach their respective borders between the sample plug and the electrolyte (terminating or leading electrolyte), the electrical field decreases and the electrophoretic velocity of cations decreases and that for anions increases (Mala et al., 2009). The stacking applied in subsequent experiments introduced an alkaline leading electrolyte with high conductivity in front of the sample (step A4, Table 1) and behind the injected sample a terminating electrolyte having low conductivity with slight acidity (step A6, Table 1). Using these optimized conditions the first standard mixtures were analyzed. In Figure 2, we demonstrate the separation of both iron redox-species at a concentration of 50 μg/L. Peak shapes were good and achieved for Fe(III) a PWb of 5 s and a nPH of 39,100 cps for Fe(II) a PWb of 8 s and a nPH of 41,200 cps. Calibration was tested up to 600 μg/L per Fe-species, providing an acceptable linearity for Fe(III), while, for Fe(II) the curve flattened significantly above 150 μg/L. Therefore, final calibration was performed only up to 150 μg/L for both species. This was considered to be sufficient since planned samples for analysis were known to have low iron concentrations. In case of higher iron concentrations were expected, samples were diluted accordingly. Five-point calibration curves were characterized by linear equations: Fe(II): y = 17.931 × + 586.86, *r*^2^ = 0.9999; Fe(III): y = 23.151 × + 37.107, *r*^2^ = 0.9951.

### Concentration Precision and Migration Time Precision

Precision of concentration measurements were determined in real-world samples, here exemplified in 1:2 diluted lysates of human neuroblastoma cells (SH-SY5Y cell line). Precision (day 1: *n* = 3, day 2: *n* = 2, overall *n* = 5) was 3.5% for Fe(II) (determined at 62 μg/L measured concentration in diluted cell lysates) or 2.2% for Fe(III) (determined at 112 μg/L measured concentration in diluted cell lysates). Migration time precision in SH-SY5Y cell lysates was 2% for Fe(III) and 3% for Fe(II) as well as for an unknown Fe-peak. It should be noted that the migration time in CZE depends aside from others, like molecule charge and size, on conductivity at sample plug, which causes migration time variation or shifts when samples themselves considerably influence conductivity. Standards and SH-SY5Y cell lysates had moderate and homogenous conductivity. Consequently, there migration times showed only small changes with good precision. However, when analyzing different samples, such as CSF samples having high salinity, conductivity was affected and migration was shifted compared to standards. Precision within that sample type again can reach good values when homogenous conductivity is present within that sample group. Nevertheless, species identification could not be performed just by migration time match, but either using internal standards or best applying standard addition. This is detailed below in section “*Proof of principle: Analysis of CSF samples.”*

### Accuracy

Currently, there are no standard reference materials available for Fe(II) or Fe(III) determination. Therefore, we checked measurement accuracy **(a)** by parallel determinations of SH-SY5Y lysate samples using the established LC-ICP-MS method (Solovyev et al., 2017) and **(b)** by recovery experiments after standard addition.

- Measurement values by evaluated LC-ICP-MS method were set to 100%. Comparison (*n* = 6) of CE-ICP-MS measurements revealed 97.6 ± 3.7% accuracy for Fe(III) and 105 ± 6.6% for Fe(II).
- Recovery of standard addition (added concentration: *(a)* = +33 μg/L or *(b)* = +500 μg/L, each per species) into real samples was *(a):* 97.2 ± 13% [Fe(II)] or 108 ± 15% [Fe(III)], 102.5 ± 7% (sum of species), and *(b)* 99 ±4% [Fe(II)] or 101 ± 6% [Fe(III)], 100 ± 5% (sum of species).

### Uncertainty

We calculated the expanded uncertainty based on a coverage factor *k* = 2 and the combined uncertainty, the latter being based on determined measurement variations of 24 SH-SY5Y cell lysate samples. These determinations included the uncertainty of the entire steps from sample preparation, automated capillary preparation for measurement, sample injection, stacking and analysis. The expanded uncertainty was 4.5% for Fe(III) and 7% for Fe(II), both determined in SH-SY5Y cell lysates.

### Limits of Detection and Quantification

Limit of detection (LOD) or limit of quantification (LOQ) were calculated according to 3σ (LOD) or 10σ (LOQ) criterion at concentration 33 μg/L per species with 13 nL sample injection volume. For Fe(III) LOD = 3.2 μg/L and LOQ = 10.8 μg/L were found. For Fe(II) LOD = 3.1 μg/L and LOQ = 10.4 μg/L were calculated. These LODs and LOQs were superior compared to all Fe(II)/(III) CE speciation analysis methods which did not use on-line coupled ICP-MS detection: Owens et al. (2000) reported LODs of 2–50 μM Fe (corresponding to 112–2,800 μg/L Fe), Gotti et al. (2015) found LODs or LOQs of 24 or 72.6 μM Fe(III) (corresponding to 1,344 μg/L or 4,065.6 μg/L) and LOD = 1.6 μM or LOQ = 4.8 μM Fe(II) (corresponding to 89.6 μg/L or 268,8 μg/L). Wilson and Carbonaro (2011) did not report LOD or LOQ, but the presented electropherograms were in the 100–200 μM range (corresponding to 5,600–11,200 μg/L range) which is far above the LODs reported here and with little baseline noise already visible. Each of those papers used UV-Vis detection of different Fe-complexes. In our previous papers using LC separation, we achieved 6.33 μg/L for Fe(III) and 9.11 μg/L for Fe(II) when using ICP-optical emission spectrometry for detection (Fernsebner et al., 2014). However, recoveries were less: for Fe(III) it was 43–66% and for Fe(II) it was 83–105%. In a further, optimized follow-up study with LC-ICP-sf-MS the LODs were calculated at 0.5 μg/L [Fe(III)] and 0.6 μg/L [Fe(II)]. Recoveries (sum of Fe-species vs. total iron determination) with this optimized LC-based method were 92 ± 11%.

### Species Stability

**Figure 3:**
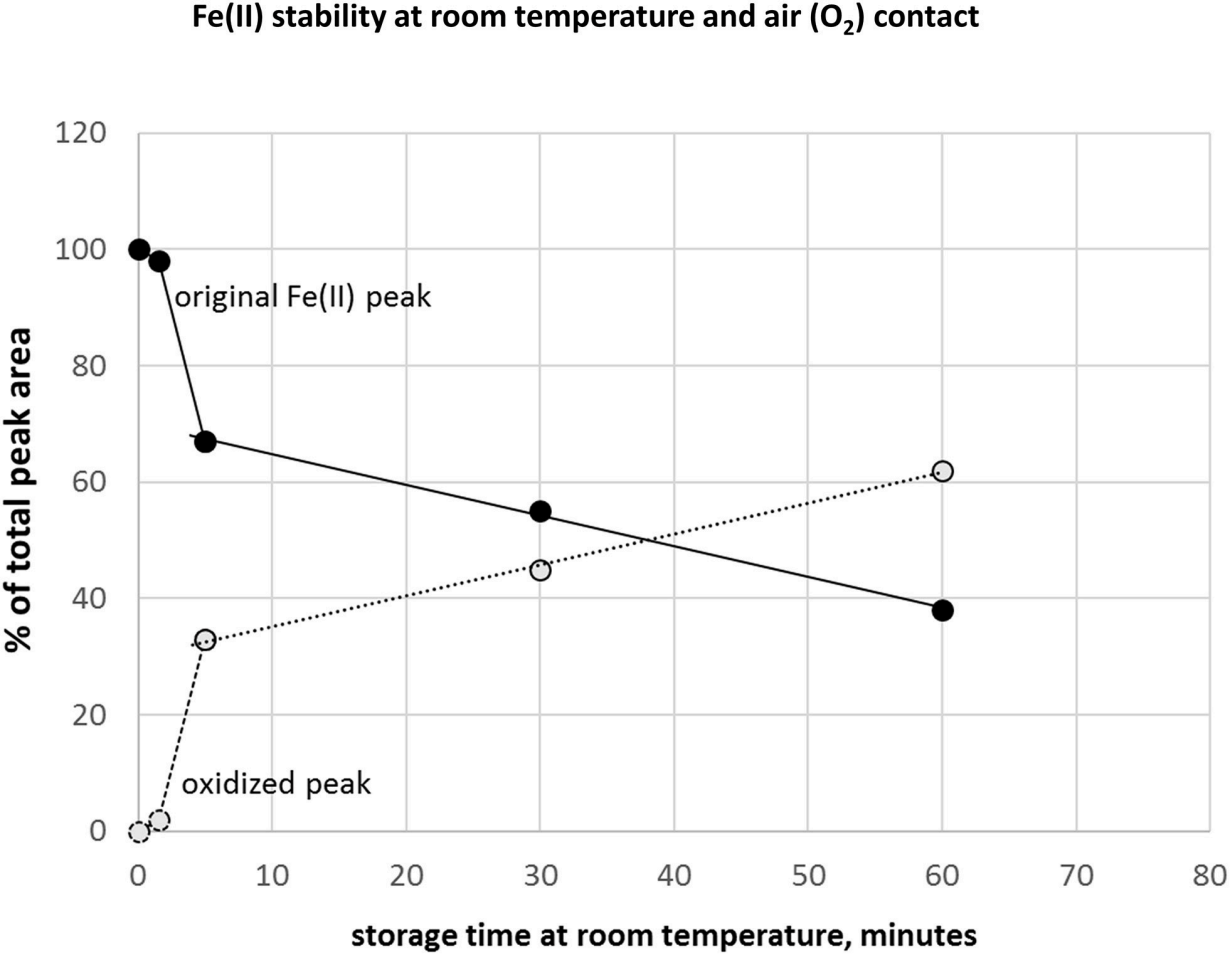
Stability of Fe(II) under inappropriate storage condition at room temperature and air (oxygen) access to sample.

A critical issue in iron redox speciation analysis is species stability. Fe(II)/(III) equilibria are easily changed under inappropriate storage conditions, such as oxygen (air) contact with sample or a break in deep-frozen storage (Fernsebner et al., 2014; Solovyev et al., 2017). Therefore, we applied an Ar overlay in the sample containers and immediately deep froze all analyzed samples. In case the analytical system is in stand-by instead of ready-to-start-measurement mode after fresh preparation of standards, additional minutes could be wasted where samples are exposed to air oxygen at room temperature. In this context, we investigated how additional storage time under sub-optimal conditions affects Fe-redox species. Figure 3 shows the time course of peak area reduction of the original Fe(II) peak and appearance of the changed species supposedly by oxidation of Fe(II), expressed as changes in % of total peak area. Analysis within 1.5 min still provided good results with 98–100% recovery of original species, but already at 5 min storage time a drop to 66% was observed. These results underlined the essential care to be considered in pre-analytical steps to maintain native speciation, specifically when analyzing iron redox species (Isai Urasa, 1993; Quevauviller et al., 1993; Solovyev et al., 2017). The stability of Fe(II) and Fe(III) in HCl had been investigated previously. When HCl concentration was increased, Fe(III) tended to be reduced to Fe(II) and further converted to a Fe-chlorocomplex (Isai Urasa, 1993). However, below 100 mM HCl concentration such conversions were reported to be negligible and quantification sensitivity by element selective detection (optical emission spectrometry) was found to be equal under these conditions for both Fe species (Isai Urasa, 1993). From those findings as well as our here presented recovery results, it can be expected that the electrolyte concentration of 20 mM HCl did not negatively affect Fe-redox speciation.

### Proof of Principle:

#### Analysis of SH-SY5Y Cell Lysates

We previously revealed that manganese (Mn) alters the Fe(II)/Fe(III) ratio toward Fe(II), and thus induces OS and LPO in neuronal cells and *in vivo* (Fernsebner et al., 2014; Venkataramani et al., 2018). Therefore, we applied our method to Mn-exposed SH-SY5Y cells cultured in OptiMEM media. Quantitative Fe(II) and Fe(III) measurements using our CE-ICP-MS method revealed an accumulation of Fe(II) resulting in a shift of the Fe(II)/Fe(III) ratio from 0.43 (±5.7%) to 0.64 (±5.2%). This ratio was similar to our previous published data with Mn-exposed SH-SY5Y cells cultured in DMEM media [Fe(II)/Fe(III) ratio from 0.4 to 0.7] (Venkataramani et al., 2018).

In conclusion, we here demonstrated that our CE-ICP-MS-based speciation analysis method can reliably detect changes in the Fe(II)/Fe(III) ratio in cell lysates and therefore also applicable to other Fe(II)-related biological contexts such as FPT.

#### Analysis of CSF Samples

**Figure 4:**
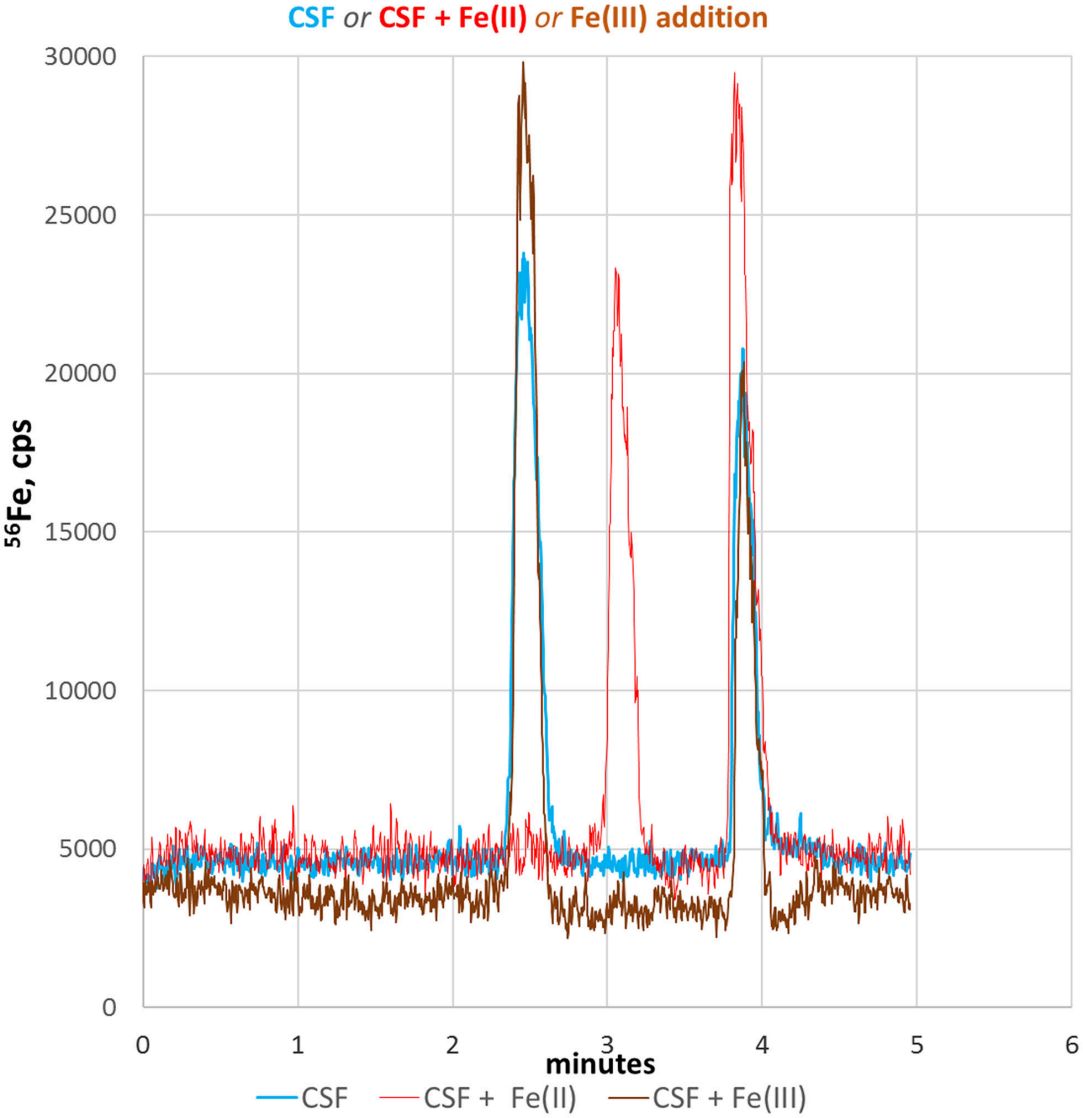
Fe-redox speciation analysis in a CSF sample (blue line). Peak identification is performed by standard addition to CSF of either Fe(II) (red line) or Fe(III) standard (brown line).

CSF is mainly an excretion of the choroid plexus in the brain ventricles and plays an important role in the metabolic homeostasis of the central nervous system. Since CSF is directly connected without a barrier to the extracellular space of brain parenchyma, CSF best reflects molecular changes of brain tissue and therefore can be utilized to monitor pathophysiological relevant fluctuations in neuronal Fe-redox balance (Agamanolis, 2016). Therefore, we applied our CE-ICP-MS method to CSF samples to demonstrate its applicability to clinical relevant samples. Figure 4 shows the respective electropherogram (blue line). Compared to standards and cell lysates, the migration times of both iron species in CSF were shifted considerably and appeared later. This might be due to the high salinity of CSF samples (up to 150 mM NaCl + other alkali salt concentrations) resulting in shifted conductivity (Harrington et al., 2010; Agamanolis, 2016). Migration time shifts under changed conductivity condition are a CZE-immanent problem (Kuhn and Hofstetter-Kuhn, 1993; Michalke, 1995). For clear identification, standard additions were performed and respective peaks increased as demonstrated in Figure 4 (red and brown lines). Such addition can again change sample conductivity which again causes variation in migration times, as investigated in Michalke (1995). The identification thus goes along with peak pattern comparison: The same (or very similar) peak pattern should be observed as in the original samples, with one specific peak being increased after addition, while the exact migration times may have changed with standard addition. The increased peak then can be considered as likely to be identified. Our experiments revealed that the addition of Fe(III) did not change the typical migration times of the redox species in CSF. However, the addition of Fe(II) considerably influenced the migration time of Fe(III), which markedly migrated slower. This could be explained by a pronounced conductivity change at sample position caused by Fe(II) addition, influencing the faster species Fe(III) more than the later eluting species Fe(II) (Kuhn and Hofstetter-Kuhn, 1993).

In conclusion, we here demonstrated that our CE-ICP-MS based method can be applied to clinically relevant biofluids, such as CSF. Moreover, the quantitative detection of Fe(II)/Fe(III) species in CSF could be valuable to monitor iron homeostasis in several neurodegenerative disorders where an altered Fe(II)/Fe(III) ratio might reflect disease progression and neuronal tissue at risk to undergo through FPT.

## Conclusion

Iron is the most abundant transition metal in the human body and plays a pivotal role in OS, LPO and FTP (Dixon et al., 2012; Stockwell et al., 2017). In order to better understand the role of Fe(II) in these contexts, several research groups successfully developed Fe(II)-specific probes to visualize the labile iron pool with spatial and temporal resolution *in vitro* and partially also *in situ*. However, all these semi-quantitative methods only can give information of either Fe(II) or Fe(III) and moreover are not applicable to biofluids, such as CSF (Ackerman et al., 2017). We here present a versatile CE-ICP-MS based quantitative method for simultaneous Fe(II)/Fe(III) speciation analysis in cell lysates and CSF. Compared to previous LC-based methods, our CE-based method not plagued with problems of batch-to-batch variability of stationary phases and generates fast reliable results with suitable figures of merit for pre- and clinical samples. Capillary preparation before each run is <4 min and analysis time per sample with moderate salinity <3 min, or for high-salt samples (CSF) <4–5 min. Carefully designed studies that take this method in account are needed to validate if Fe(II)/Fe(III) quantification can serve as potential biomarker for neurodegenerative disease progression and may also serve to pinpoint tissues at risk for Fe(II)-mediated lethal damage.

## Data Availability

All datasets generated for this study are included in the manuscript and/or the supplementary files.

## Author Contributions

BM performed the stepwise CE-ICP-MS development measurements and performed measurements and calculations for characterizing the figures of merit of the method. DW performed LC-ICP-MS measurements for accuracy determinations and measurements of CSF samples. VV prepared and characterized SH-SY5Y cell lysates. All authors prepared the manuscript.

### Conflict of Interest Statement

The authors declare that the research was conducted in the absence of any commercial or financial relationships that could be construed as a potential conflict of interest.

## Abbreviations

CE: capillary electrophoresis
CSF: cerebrospinal fluid
CZE: capillary zone electrophoresis
DRC: dynamic reaction cell
FPT: ferroptosis
ICP-MS: inductively-coupled-plasma-mass-spectrometry
LC: liquid chromatography
LOD: limit of detection
LOQ: limit of quantification
LPO: lipid peroxidation
nPH: net peak height
OS: oxidative stress
PBS: phosphate buffered saline
PW_b_: peak width at baseline
PDCA: 2,6-pyridine dicarboxylic acid
ROS: reactive oxygen species
TMAH: tetramethyammoniumhydroxide.
